# Pesticide-tolerant bacteria isolated from a biopurification system to remove commonly used pesticides to protect water resources

**DOI:** 10.1371/journal.pone.0234865

**Published:** 2020-06-29

**Authors:** Gabriela Briceño, Claudio Lamilla, Bárbara Leiva, Marcela Levio, Pamela Donoso-Piñol, Heidi Schalchli, Felipe Gallardo, María Cristina Diez

**Affiliations:** 1 Biotechnological Research Center Applied to the Environment (CIBAMA-BIOREN), University of La Frontera, Temuco, Chile; 2 Chemical Sciences and Natural Resource Department, University of La Frontera, Temuco, Chile; 3 Chemical Engineering Department, University of La Frontera, Temuco, Chile; Wilfrid Laurier University, CANADA

## Abstract

In this study, we selected and characterized different pesticide-tolerant bacteria isolated from a biomixture of a biopurification system that had received continuous applications of a pesticides mixture. The amplicon analysis of biomixture reported that the phyla Proteobacteria, Firmicutes, Bacteroidetes and Actinobacteria were predominant. Six strains grew in the presence of chlorpyrifos and iprodione. Biochemical characterization showed that all isolates were positive for esterase, acid phosphatase, among others, and they were identified as *Pseudomonas*, *Rhodococcus* and *Achromobacter* based on molecular and proteomic analysis. Bacterial growth decreased as both pesticide concentrations increased from 10 to 100 mg L^-1^ in liquid culture. The *Achromobacter* sp. strain C1 showed the best chlorpyrifos removal rate of 0.072–0.147 d^-1^ a half-life of 4.7–9.7 d and a maximum metabolite concentration of 2.10 mg L^-1^ at 120 h. On the other hand, *Pseudomonas* sp. strain C9 showed the highest iprodione removal rate of 0.100–0.193 d^-1^ a half-life of 4–7 d and maximum metabolite concentration of 0.95 mg L^-1^ at 48 h. The *Achromobacter* and *Pseudomonas* strains showed a good potential as chlorpyrifos and iprodione-degrading bacteria.

## Introduction

Different pesticides are applied simultaneously in the field to improve the effectiveness of pest control, thereby increasing environmental contamination and affecting the soil and water quality [1,2]. To minimize pesticide point-source contamination, a preventative technology of biopurification named biobed, was introduced and implemented in Sweden in the 1990s by Torstensson and Castillo [3] to reduce the risk of water resource contamination. Pesticide removal by this biopurification technology is based on the adsorption and degradation capacity of an organic, biologically active biomixture that is composed of topsoil, peat, and lignocellulosic material cover by a vegetal layer [4,5]. The biopurification system (BPS) is highly efficient in pesticide removal, achieving high degradation of different pesticides that are commonly applied in farms, even after repeated applications [6,7].

Microbial communities are considered to be key factors in controlling the depuration capacity of BPSs, and knowledge of the biological activity occurring in the biomixture is relevant for understanding pesticide degradation and for optimizing their degradation [7–9]. In this respect, some studies have correlated pesticides degradation in biomixtures with microbial activities, such as phenoloxidase activity [10–12], the respiration rate [13], and microbial community changes [6, 14–15]. A genotypically and phenotypically versatile microbial community can degrade different pesticide residues at different concentrations in a biomixture [6]. Greater bacterial diversity compared to fungal diversity has been reported such that bacterial diversity increased throughout the biopurification system, as was induced by pesticide exposure [16].

It is well-known that microorganisms are responsible for the degradation of pesticides in soils [17,18]. This degradation is due to the extensive use of these compounds in agricultural soils, which has induced mechanisms of genetic adaptation in microorganisms. These genetic adaptations have led to the synthesis of enzymes that oxidize, hydrolyse, and hydroxylate pesticides, which allows them to use pesticides as the sole source of carbon, nitrogen, sulfur, or phosphorus and facilitates the elimination of the compound's toxicity [19]. Furthermore, active microbial populations develop in the soil with the ability to degrade persistent compounds after repeated pesticide application in the same field for a certain number of years [20].

Although bacterial species play important roles in the transformation of pesticides, complete mineralization of pesticide residues is more likely to occur with mixed populations than with individual microorganisms [21]. Among the bacteria responsible for the degradation of pesticides, the genera *Streptomyces*, *Arthrobacter*, and *Achromobacter* have been isolated from contaminated soil and from soil with historical application and have been studied due to their strong capacity to degrade various pesticide residues, including chlorpyrifos (CHL) and iprodione (IPR) [22–25]. Campos et al. [24] reported two strains, *Arthrobacter* sp. strain C1 and *Achromobacter* sp. strain C2 isolated from soil, which are able to transform IPR and its degradation metabolite, 3,5-dichloraniline (3,5-DCA), in different culture media. Additionally, metabolic intermediates 3,5-dichlorophenyl-carboxamide and 3,5-dichlorophenylurea-acetate in the metabolic IPR pathway, produced by these soil bacteria and their combination, were reported by Campos et al. [9]. Briceño et al. [22] reported two *Actinobacteria* isolated from an agricultural soil that had received continuous applications of CHL, which were able to degrade this compound rapidly with approximately 90% degradation after a 24-h incubation.

Furthermore, several studies have reported the potential of indigenous microbial consortia isolated from contaminated soils to degrade different pesticides and pesticide mixtures. In this context, Fuentes et al. [26] reported a *Streptomyces* sp. consortium that can remove an organochlorine pesticide mixture composed of lindane, methoxychlor, and chlordane. Recently, mixed cultures of the fungus *Trametes versicolor* and the bacteria *Streptomyces* spp. were used to inoculate different biomixtures based on their previously demonstrated ligninolytic and pesticide-degrading activities [21]. The authors demonstrated that the consortium improved lindane dissipation (81–87%) or removal after 66 d of incubation in different biomixtures, thereby decreasing the lindane half-life to an average of 24 d, which is 6-fold less than the half-life of lindane in soils. In addition, Briceño et al. [27] reported for the first time the removal of an organophosphorus pesticide mixture composed of CHL and diazinon from different environmental matrices, including liquid medium, soil, and a biobed biomixture, by using a *Streptomyces* mixed culture.

The previous studies mentioned above have reported the ability of selected bacteria isolated from pesticide-contaminated soils to remove pesticides. However, the isolation and characterization of pesticide-degrading microorganisms from a BPS used for pesticide treatment have been rarely studied. We hypothesize that in a system that is continuously receiving a load of different pesticides, it would be possible to find tolerant and adapted microorganisms that are capable of degrading pesticides at high concentrations. Therefore, the goal of this study was to select and characterize bacterial species isolated from a BPS that have the ability to degrade the fungicide IPR and the insecticide CHL.

## Materials and methods

### Pesticides and culture media

Analytical grade (99%) IPR, 3,5-DCA, CHL, and 3,5,6-trichlo-2-pyridinol (TCP) for chromatographic analyses by HPLC were purchased from Sigma-Aldrich (St. Louis, MO). The stock solutions (1,000 mg L^−1^) in acetone were sterilized by filtration through 0.22-μm pore-size membranes. For degradation assays, formulated commercial CHL (Troya 4EC) and IPR (Rovral 50 WP) were purchased from Agan Chemicals Manufacturers Ltd (S1 Table). Commercial products were prepared individually in a stock solution of 10,000 mg L^-1^ in methanol, filtered through a 0.22-mm PTFE filter, and then stored at 4°C until their use. All other chemicals and solvents were of analytical reagent grade (Merck).

Mineral salts medium (MSM) broth containing (per L) 1.6 g K_2_HPO_4_, 0.4 g KH_2_PO_4_, 0.2 g MgSO_4_ · 7H_2_O, 0.1 g NaCl, 0.02 g CaCl_2_, and 1 mL salt stock solution (2.0 g boric acid, 1.8 g MnSO_4_ · H_2_O, 0.2 g ZnSO_4_, 0.1 g CuSO_4_, 0.25 g Na_2_MoO_4_, 1000 mL distilled water) was used for isolation of pesticide-tolerant bacteria. The initial pH of the medium was adjusted to 7.0 prior to sterilization by autoclaving at 121°C for 20 min. Subsequently, 0.05 g L^-1^ cycloheximide was added to avoid fungal contamination. Luria Bertani (LB) broth containing (per L) 5.0 g NaCl, 2.5 g yeast extract, and 10.0 g casein peptone was used for routine cultivation of the isolated bacteria and for degradation assays. The pH of LB was adjusted to 7.0 prior to autoclaving. Plate count agar (PCA) containing (per L) 5.0 g tryptone, 2.5 g yeast extract, 1.0 g glucose, 15.0 g agar-agar was adjusted to pH 7.2 prior to sterilization, and 0.05 g cycloheximide was added to avoid fungal contamination. The R2A agar containing (per L) 0.5 g casein acid hydrolysate, 0.5 g yeast extract, 0.5 proteose peptone, 0.5 g dextrose, 0.5 g soluble starch, 0.3 g K_2_HPO_4_, 0.024 g MgSO_4_, 0.3 g sodium pyruvate, and 15 g agar, pH 7.2 commonly used for heterotrophic bacteria was used for strains biochemical characterization.

### Biomixture sampling

Biomixture sample was obtained from a BPS used during the last three years for the treatment of a mixture of pesticides, including CHL and IPR at 50 mg kg^-1^ a.i. each, with re-application every 30 d [5]. The BPS consisted of a plastic tank of 1 m^3^ capacity packed with 125 kg biomixture (dry weight) (bulk density (ρ) 0.29 g mL^-1^), which reached a height of 60 cm. The biomixture was prepared with topsoil, commercial peat, and wheat straw in a proportion of 1:1:2 (v v^-1^), and the humidity of biomixture was maintained at approximately 65–70% of water-holding capacity (WHC) by the addition of tap water. For strains isolation, selection and characterization, samples were taken from different sections of the BPS, placed in a sterile plastic bag, homogenized and stored at 4°C for no longer than 12 h until their use.

### Microbial community composition of biomixture

The microbial community composition in the biomixture was analyzed by using a MinION™ portable nanopore sequencer. Total community DNA was extracted from 0.25 g of the homogenized sample of the biomixture acquired in the previous activity by using MoBio Powersoil DNA isolation kit (MoBio, Carlsbad, CA) following the manufacturer's instructions. The 16S rRNA gene library was prepared according to the manufacturer’s instructions and sequencing was carried out on a MinION sequencer (ONT) using an R9.4 flowcell. Amplification of the 16S rRNA gene was carried out with the 16S Barcoding kit SQK-RAB204 of Oxford Nanopore Technologies using the Master mix LongAmp Taq 2X (NEB) following the instructions provided by Oxford Nanopore Technologies. PCR products were purified with the AMPure XP beads kit (Beckman Coulter, Brea, CA, USA) and quantified using the Qubit dsDNA BR Assay kit (Invitrogen, Merelbeke, Belgium) with a Qubit 2.0 Fluorimeter. To optimize the output of the MinION platform, a multiplexing strategy was applied using the 16S Barcoding kit SQK-RAB204 (ONT).

### Isolation and selection of pesticide-tolerant bacteria

For pesticide-tolerant bacteria isolation, 10 g of the homogenized biomixture obtained in biomixture sampling activity were put into 250 mL Erlenmeyer flasks containing 90 mL of MSM broth supplemented with a pesticide mixture composed of CHL and IPR at 10 mg L^-1^ a.i. each. The flasks were incubated for 7 d at 28 ± 2°C and 130 rpm with constant shaking. A tenfold dilution series was prepared and 100 μL aliquots were placed on Petri dishes containing 30 mL of PCA medium and incubated at 28 ± 2°C for 7 d. Each bacterial colony displaying different characteristics was isolated and maintained on LB-glycerol (70/30%) medium slants at -80°C.

For bacterial inocula preparation, isolated strains were re-activated on plate dishes with PCA medium, incubated for 48 h at 28 ± 2°C. Then, bacteria were grown in 100 mL flasks with LB broth at 28 ± 2°C for 48 h and 130 rpm in a rotatory shaker. The biomass was collected by centrifugation at 6000 rpm for 10 min, and the cell pellets were then washed with sterile NaCl solution (0.9%). The LB medium was used instead of the MSM medium due to the low growth that had been observed for isolated strains cultured in MSM.

For pesticide-tolerant bacteria selection, biomass growth in LB broth supplemented with pesticides was evaluated. Flasks containing 50 mL of LB broth and a mixture of CHL and IPR at 10 mg L^-1^ concentration each were inoculated with 1% (v v^-1^) of each isolated strains. Flasks were incubated at 28 ± 2°C and 130 rpm under constant shaking during 48 h. Bacterial growth was evaluated by absorbance at 600 nm. The absorbance values were converted into biomass dry weight (g L^-1^) by using a calibration curve (R^2^ > 0.999). Flasks without the pesticides were used as biotic controls. The assay was conducted in triplicate.

### Characterization of selected pesticide-tolerant strains

The selected bacteria strains that showed a biomass concentration ≥ 1.0 g L^-1^ in LB broth supplemented with the pesticide mixture were characterized by a combination of phenotypic tests that were based on the colony morphology, gram-staining reaction, and colony pigmentation [28]. Cellular visualization of pesticide-tolerant bacteria was performed by using scanning electron microscopy with variable pressure (VP-SEM) (SU-3500 Hitachi-Japan). A 65-μL re-activated sample of each strain were placed in the equipment sampler and was dried at 30°C for microscopic observations by SEM.

Biochemical characterization using the APIZYM kit (Biomerieux, France) according to the manufacturer's instructions was made for selected strains. This microbial identification system consists of 19 substrates in a microplate, which was incubated at 28°C for up to 4 d. The enzymatic reaction was detected based on the intensity of the color that developed following the addition of reagents. Moreover, extracellular hydrolytic enzyme production was screened as described by Margesin et al. [29]. The presence of amylase, cellulase, lipase, protease, and gelatinase activity was tested on R2A agar supplemented with starch (0.4% w v^-1^), carboxymethylcellulose and trypan blue (0.4% and 0.01% w v^-1^), Tween 80 (1% v v^-1^), skim milk powder (0.4% w v^-1^), or gelatine (1% w v^-1^), respectively. Plates were incubated between 3 to 10 d at 15°C, depending on the enzyme being analyzed. A reaction was considered to be positive when the transparent zones around the colonies were visible or detected after precipitation or coloration of the non-degraded substrate. To reveal the amylase and protease activities, the plates were stained with Lugol’s solution and Coomassie brilliant blue solution, respectively [29]. For all the assays, the agar plates were prepared in triplicate.

### Bacterial identification by sequence analyses and MALDI-TOF/TOF MS

For identification of the selected pesticide-tolerant bacteria, genomic DNA was extracted using the UltraClean® Microbial DNA Isolation Kit (MOBIO, CA, USA) according to the manufacturer’s instructions. 16S rRNA genes were selectively amplified from genomic DNA by polymerase chain reaction (PCR) using universal primers 27F (5’-AGAGTTTGATCCTGGCTCAG-3’) and 1492R (5’-GGTTACCTTGTTACGACTT-3’), enabling the amplification of approximately 1,500 bp of the 16S rRNA gene. PCR amplification was performed in a Multigene Optimal Thermal Cycler (Labnet, USA) in 50 μL of PCR mix comprising 25 μL mix reaction buffer 2x (SapphireAmp Fast PCR Master Mix, Takara), 22 μL ultra-pure water, 1 μL of each primer (10 μM), and 1 μL of DNA. The temperature and cycling conditions were as follows: preheating at 94 ºC for 2 min; 30 cycles at 94 ºC for 1 min; 55 ºC for 1 min; 72 ºC for 1.5 min; and incubation at 72 ºC for 10 min. The presence of PCR products was assessed by electrophoresis on a 1% agarose gel stained with gel red. Sequencing was conducted using a dye Terminator Cycle Sequencing Kit and an ABI 3730XL DNA Sequencer (Applied Biosystems) by Macrogen (Korea). The nearest taxonomic group was identified by 16S rRNA nucleotide sequence BLASTN (http://www.ncbi.nlm.nih.gov/blast) using DDBJ/EMBL/GenBank nucleotide sequence databases. The phylogenetic affiliation of bacteria in GenBank was performed using MEGA7.

For the MALDI-TOF/TOF MS analysis, samples of the selected bacterial colonies were applied directly to the equipment sampler plate and were coated with a saturated solution of α-cyano 4-hydroxy cinnamic acid diluted in 50% acetonitrile with 2.5% trifluoracetic acid. Mass spectra were obtained by using a MALDI-TOF/TOF MS Autoflex Speed (Bruker Daltonics, Bremen, Germany) equipped with a smart beam laser source (334 nm). Analyses were performed in linear mode with positive polarity, 20 kV acceleration voltage, and extraction with a 220-ns delay. Each spectrum was collected as an average of 1200 laser shots with enough energy to produce good spectra without saturation in the range of 2000 to 20,000 m/z. Analyses equipment was calibrated externally by using the protein calibration standard I (insulin, ubiquitin, cytochrome C and myoglobin) with Flex Control 1.4 software (Bruker Daltonics, Bremen, Germany). The sample analyses were performed with the MALDI Biotyper Compass 4.1 software (Bruker Daltonics, Bremen, Germany) in the range of 3,000–15,000 m/z, compared with a library of 6509 spectra of bacterial identifications. According to the guidelines of the manufacturer, a score of ≥ 2 depicts identification to the species level, and an intermediate log score between < 2 and ≥ 1.7 for identification to the genus level. A dendrogram generated by MALDI Biotyper mass spectra was performed for all strains isolated after enrichment with CHL and IPR in liquid cultures.

### Pesticide degradation

The pesticides degradation assay was conducted in 250 mL flasks containing 100 mL LB broth supplemented with each individual pesticide at increasing concentrations of 0, 10, 20, 50, and 100 mg L^-1^. Flasks were inoculated with 1% (v v^-1^) of each selected strains. Flasks without pesticides and non-inoculated flasks were run as biotic and abiotic controls, respectively. Subsequently, the flasks were incubated at 28 ± 2°C on a rotary shaker at 130 rpm for 48 h and 120 h for IPR and CHL, respectively. Samples were taken at different times for analyses of bacterial biomass, pesticide concentration, and pesticide metabolite by high-performance liquid chromatography (HPLC). For biomass growth and pesticide degradation, the kinetics parameters were calculated. All assays were conducted in triplicate.

### Analyses of pesticides and metabolites

Samples (1 mL) were taken from each flask, and centrifuged at 6500 rpm for 10 min. After that, 0.5 mL of the supernatant was diluted in 1 mL of acetonitrile grade HPLC. The samples were homogenized in a vortex and filtered by PTFE 0.22 μm filter before analysis of pesticide concentrations. Analysis was performed using a Merck Hitachi L-2130 pump equipped with a Rheodyne 7725 injector and a Merck Hitachi L-2455 diode array detector. Separation was achieved using a C18 column (Chromolit RP-8e, 4.6 μm × 100 mm). The mobile phase was 70% 1 mM ammonium acetate and 30% acetonitrile injected at a flow rate of 1 mL min^-1^. The column temperature was maintained at 30 ± 1°C; the detector was set for data acquisition at 290 nm. Instrument calibrations and quantifications were performed against pure reference standards (0.01–10 mg L^-1^) for each pesticide. Average recoveries for the pesticide were: IPR, 92 ± 2.2%; CHL, 101 ± 0.7%. Limit of quantification (LOQ) was determined using the smallest concentration of the analyte in the test sample, which induced a signal that was ten times higher than the background noise level (CHL = 0.214 mg L^-1^ and IPR = 0.238 mg L^-1^). Limit of detection (LOD) was 0.081 mg L^-1^ for CHL and 0.089 mg L^-1^ for IPR.

### Kinetics and statistical analysis

The degradation assay data was used to determine the specific growth rate for the exponential phase by using the following equation:

$$\mu max=\frac{dx}{dt}\times \frac{1}{x}$$

where μ = specific growth rate (h^-1^), x = biomass concentration (g L^-1^), and t = time (h). The removal of CHL and IPR was described by using the first-order kinetic model:

$$Removal=\ln\frac{Ct}{Co}e^{-kt}$$

where C_0_ is the amount of contaminant in the liquid medium at time zero, C_t_ is the amount of contaminant at time t, and k and t are the rate constant and degradation time in hours, respectively. The time at which the CHL and IPR concentrations in the liquid medium were reduced by 50% (T_1/2_) was calculated by using the following equation:

T1/2=ln(2)/k.

A simple correlation analysis was performed to determine the correlation between the biomass growth and the initial pesticide concentration. The data were averaged, and the standard deviation (SD) of the means was calculated. Removal percentage data were transformed by using an angular transformation (arc sen √x/100) prior to statistical analysis. A post hoc analysis of the differences in means of the assay data was conducted with the Tukey test (α = 0.05). Statistical analyses were performed using SPSS statistical software version 17.

## Results

### Microbial composition of biomixture

**Fig 1:**
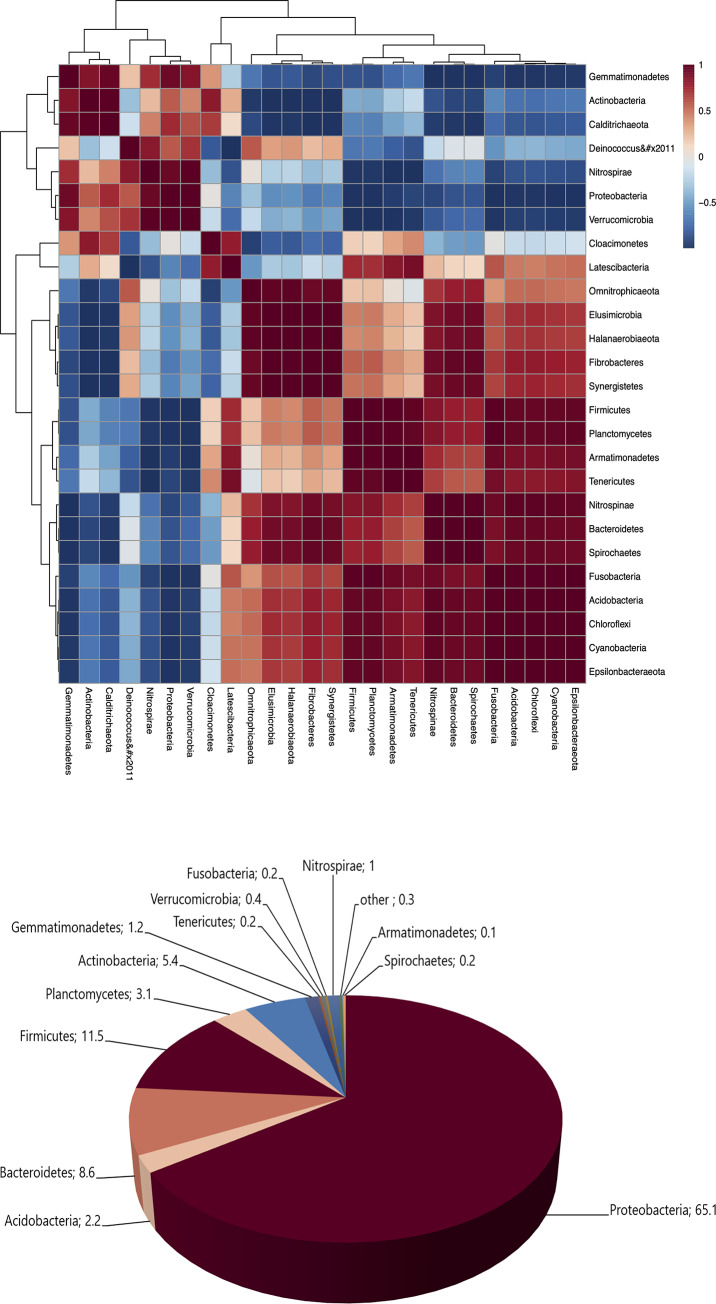
**(a)** Heatmap of Spearman's correlation coefficients between relative abundance of bacterial phyla in the biomixture. The colors of the boxes indicate the value of correlation coefficients. Darkest red indicates a perfect positive correlation (r = 1), darkest blue indicates perfect negative correlation (r = -1), and fading colors indicate a gradual loss in correlation. **(b)** Classification of bacterial community of biomixture in term of relative abundance.

According to biodiversity analysis with the obtained sequences clustered into 154,775 operational taxonomic units at 97% similarity, 26 bacterial phyla were assigned and identified in the biomixture treated with repeated pesticide applications. Spearman´s correlation analysis of phyla was made similarly to Chen et al. [30]. In the biomixture analysis indicated the positive (red) or negative (blue) correlation between bacterial phyla (Fig 1A). In general, bacteria belonging from Proteobacteria phylum presents coexistence with 7 phyla including the Actinobacteria phylum but not with Bacteroidetes phyla. The dominant Phyla composition of the biomixture were Proteobacteria (65.1%), Firmicutes (11.5%), Bacteroidetes (8.6%) and Actinobacteria (5.4%) (Fig 1B).

### Isolated bacteria from the biopurification system

Pesticide-tolerant bacteria were isolated from a biomixture used in a BPS, which had been used in the last three years to remove a mixture of pesticides that was added repeatedly at a concentration of 50 mg L^-1^ [5]. From this biomixture, 10 different types of bacterial isolates, consecutively named as strains C1 to C10, were obtained after the enrichment of MSM supplemented with a pesticide mixture composed of CHL and IPR at 10 mg L^-1^ a.i. each. Out of the 10 bacterial colonies, only six strains presented growth expressed as the biomass concentration ≥ 1.0 g L^-1^ in LB broth supplemented with the pesticide mixture. Specifically, strains C1, C4 and C10 showed biomass growth between 1.66–2.32 g L^-1^, and strains C7, C8, and C9 showed biomass growth between 1.60–1.95 g L^-1^, whereas strains C2, C3, C5 and C6 showed biomass growth below these values; therefore, these strains were eliminated for future assays.

### Characterization of pesticide-tolerant bacteria

The strains that were selected for their tolerance and capacity to grow in the presence of pesticides were characterized based on some phenotypic and biochemical characteristics. According to gram-staining analyses, all strains, except strain C8, were Gram-negative. Most isolates exhibited cream-colored colonies; strain C8 was dark-cream colored, and strain C7 had white colonies. The morphological characteristics of the bacteria were evaluated by SEM. Five of the six selected tolerant strains were bacillus, while strain C8 presented a coccus shape. In general, strains C4 (a) and C10 (c) with a bacillus cell shape and size ranging from 0.82 × 2.35 μm to 0.91 × 1.84 μm were observed, respectively. The strains C1, C7 and C9 were omitted from SEM image due their morphological similarity with the strains C4 and C10. For strain C8 (b) with a coccus cell shape, the sizes ranged from 0.76 to 1.26 μm in diameter (S1 Fig).

Biochemical and enzymatic characterization of selected strains by API ZYM test was made. All the strains were positive for esterase-(C4), leucine aminopeptidase, acid phosphatase, and naphthol-AS-BI-phosphohydrolase. Enzymes, such as alkaline phosphatase and valine aminopeptidase, were positive in 5 of the 6 strains, esterase lipase-(C8) in 3 strains, and lipase-(C14) and trypsin in 2 strains. Strain C8 was positive for most of the enzymes involved in glucose metabolism, including α-glucosidase and β-glucosidase. Finally, the C4 and C9 strains produced five extracellular enzymes on the solid R2A medium. Regarding the production of lipases and amylase, the six selected strains were positive for both enzymes, while strains C1, C4, C8, and C9 were positive for cellulolytic enzymes (S2 Table).

### Molecular and proteomic identification of bacteria

The identification of selected strains made by both 16S rRNA sequencing and MALDI-TOF/TOF MS showed similar results. The strains that were selected for their tolerance and ability to grow in the presence of pesticides CHL and IPR were identified based on 16S rRNA sequence analysis as bacteria belonging to the phylum *Proteobacteria*, family *Alcaligenaceae*, genus *Achromobacter* (strains C1, C7, and C10), and family *Pseudomonadaceae*, genus *Pseudomonas* (strains C4 and C9). Moreover, the phylum *Actinobacteria*, family *Nocardiaceae*, genus *Rodococcus* (strain C8) was identified. Table 1 shows a comparison of the 16S rRNA sequences (entire sequence compared with available sequences in GenBank) of strains C1, C4, C7, C8, C9, and C10, which showed ≥ 96% similarity to *Achromobacter spanius*, *Pseudomonas rhodesiae*, *Achromobacter deleyi*, *Rhodococcus jialingiae*, *Pseudomonas marginalis*, and *Achromobacter kerstersii*, respectively. To identify the phylogeny of the isolates, strains from different genera were chosen to construct the phylogenetic tree. The phylogenetic analysis (S2 Fig) based on the 16S rRNA using MEGA7 software indicated that the isolates had higher similarity with the 16S rRNA sequence from pesticide-degrading bacteria, i.e., *Pseudomonas caspiana* (strains C4 and C9), *Rhodococcus jialingiae* (strain C8), and *Achromobacter spirinitus* (C1, C7 and C10).

**Table 1:** Phylogenetic assignment of isolated strains tolerant to chlorpyrifos (CHL) and iprodione (IPR) and their best match results with 16S rRNA gene sequences and MALDI–TOF/TOF BioTyper.

Strains	Most closely related strain (NCBI accession no.)^a^	Identity (%)	Acession n^o^	MALDI Biotyper database	Score^b^
**C1**	*Achromobacter spanius* (MF624722.1)	96	MK110041	*Achromobacter* sp.	2.36
**C4**	*Pseudomonas rhodesiae* (R_024911.1)	99	MK110043	*Pseudomonas* sp.	2.31
**C7**	*Achromobacter deleyi* (NR_152014.1)	97	MK110044	*Achromobacter* sp.	2.06
**C8**	*Rhodococcus jialingiae* (NR_115708.1)	98	MK110045	*Rhodococcus* sp.	2.54
**C9**	*Pseudomonas marginalis* (NR_117821.1)	99	MK110046	*Pseudomonas* sp.	2.14
**C10**	*Achromobacter kerstersii* (NR_152015.1)	98	MK110047	*Achromobacter* sp.	2.04

(a) Based on partial sequencing of 16S rRNA gene and comparison with those present in GenBank database from National Center for Biotechnology Information (NCBI) by using BLAST.

(b) Log score value of the MALDI-TOF MS identification.

The direct analysis of intact cells by MALDI-TOF/TOF MS in Table 1 showed a good spectral quality with a score identification of 2.06 to 2.54, which safely allows for accurate identification to the genus level. Genus identification of the different strains was in agreement with the 16S rRNA sequence identification. According to the dendrogram, constructed by MALDI Biotyper data of the six bacteria in the presence of CHL and IPR, *Achromobacter* sp. strains C1, C7, and C10 were differentiated and grouped separately when exposed to different pesticides. Similar response was observed for *Pseudomonas* sp. strains C4 and C9 (S3 Fig).

### Biomass growth and degradation of pesticides in liquid culture

**Fig 2:**
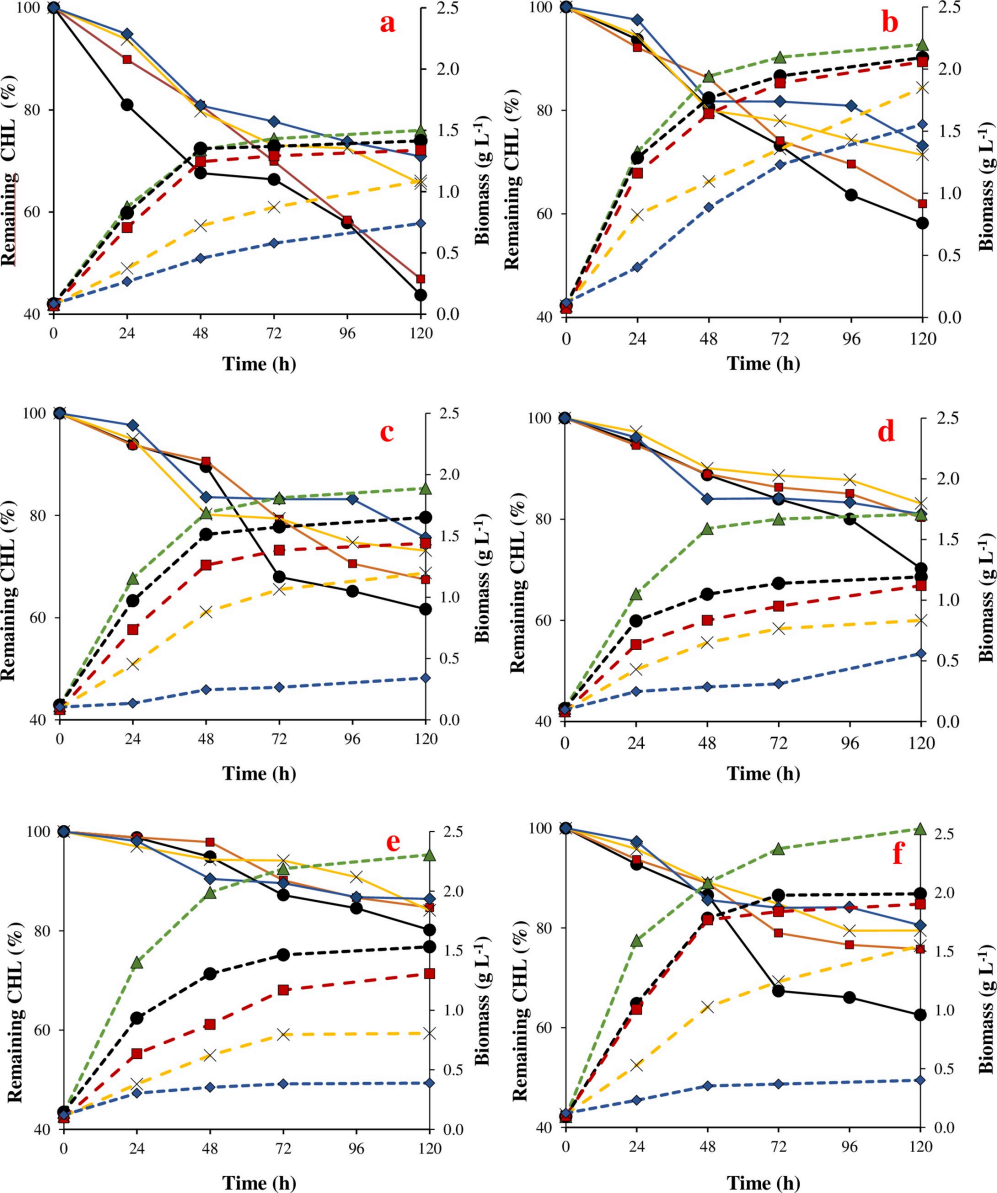
Remaining chlorpyrifos (CHL) (%) and biomass by C1 (a), C4 (b), C7 (c), C8 (d), C9 (3), C10 (f) strains at initial CHL concentration of 0, 10, 20, 50 and 100 mg L^-1^, evaluated during 120 h. (●) CHL 10 mg L^-1^; (◼) CHL 20 mg L^-1^; (x) CHL 50 mg L^-1^; (◆) CHL 100 mg L^-1^; (▲) Control without CHL. Continuous line: Remaining CHL (%); Dotted line: biomass (g L^-1^).

Biomass growth of the six tolerant-pesticide strains was evaluated at different incubation times and increasing pesticide concentrations. Bacterial growth decreased proportionally (R^2^ > 0.96) in most cases, such as the concentration of pesticides increasing from 10 to 100 mg L^-1^. Fig 2 shows that for all bacteria exposed to different CHL concentrations, the biomass increased over time. However, a high inhibition of biomass growth was observed in all the strains cultivated in liquid medium with addition of 100 mg L^-1^ CHL. *Achromobacter* sp. strain C1 and *Pseudomonas* sp. strain C4 showed the highest tolerance to 100 mg L^-1^ of CHL, with biomass over 0.5 and 1.5 mg L^-1^, respectively, at the final period of incubation.

The μ max for the studied strains ranged from 0.18 to 0.48 h^-1^ in the treatment without CHL (control treatment), and these values decreased with the increment of pesticide concentrations, showing a μ max between 0.02 to 0.16 h^-1^ for CHL added at the highest concentration (Table 2).

**Table 2:** Kinetics of bacterial growth in liquid medium contaminated with 0–100 mg L^-1^ of chlorpyrifos (CHL) and iprodione (IPR) individually.

	**Initial CHL concentration (mg L**^**-1**^**)**									
**Strain**	**0**		**10**		**20**		**50**		**100**	
	Biomass (g L^-1^)	μ max (h^-1^)	Biomass (g L^-1^)	μ max (h^-1^)	Biomass (g L^-1^)	μ max (h^-1^)	Biomass (g L^-1^)	μ max (h^-1^)	Biomass (g L^-1^)	μ max (h^-1^)
**C1**	1.50±0.10	0.18	1.41±0.00	0.17	1.34±0.02	0.16	1.09±0.03	0.11	0.74±0.05	0.09
**C4**	2.20±0.10	0.31	2.09±0.01	0.30	2.06±0.09	0.29	1.85±0.07	0.25	1.55±0.05	0.16
**C7**	1.90±0.12	0.28	1.65±0.01	0.26	1.44±0.04	0.22	1.20±0.00	0.16	0.34±0.00	0.02
**C8**	1.71±0.15	0.25	1.19±0.02	0.22	1.12±0.01	0.19	0.83±0.01	0.15	0.56±0.02	0.08
**C9**	2.31±0.00	0.36	1.53±0.00	0.28	1.31±0.00	0.22	0.81±0.02	0.14	0.39±0.04	0.11
**C10**	2.55±0.08	0.48	1.99±0.01	0.40	1.90±0.02	0.39	1.55±0.00	0.27	0.40±0.00	0.14
	**Initial IPR concentration (mg L**^**-1**^**)**									
**Strain**	**0**		**10**		**20**		**50**		**100**	
	Biomass (g L^-1^)	μ max (h^-1^)	Biomass (g L^-1^)	μ max (h^-1^)	Biomass (g L^-1^)	μ max (h^-1^)	Biomass (g L^-1^)	μ max (h^-1^)	Biomass (g L^-1^)	μ max (h^-1^)
**C1**	1.43±0.08	0.18	1.34±0.01	0.18	1.29±0.01	0.17	1.02±0.01	0.15	0.75±0.25	0.11
**C4**	2.10±0.15	0.31	1.72±0.08	0.26	1.22±0.01	0.23	1.08±0.01	0.22	0.59±0.15	0.09
**C7**	1.81±0.01	0.28	1.18±0.02	0.25	1.00±0.02	0.20	0.73±0.00	0.17	0.34±0.18	0.05
**C8**	1.67±0.01	0.25	1.48±0.04	0.24	1.24±0.00	0.22	1.05±0.02	0.20	0.56±0.08	0.12
**C9**	2.19±0.00	0.36	2.08±0.00	0.35	1.97±0.00	0.34	1.80±0.06	0.32	1.06±0.02	0.22
**C10**	2.38±0.00	0.48	1.87±0.12	0.43	1.83±0.00	0.42	1.30±0.01	0.36	0.70±0.02	0.24

The average values and the standard error are presented (n = 3)

**Fig 3:**
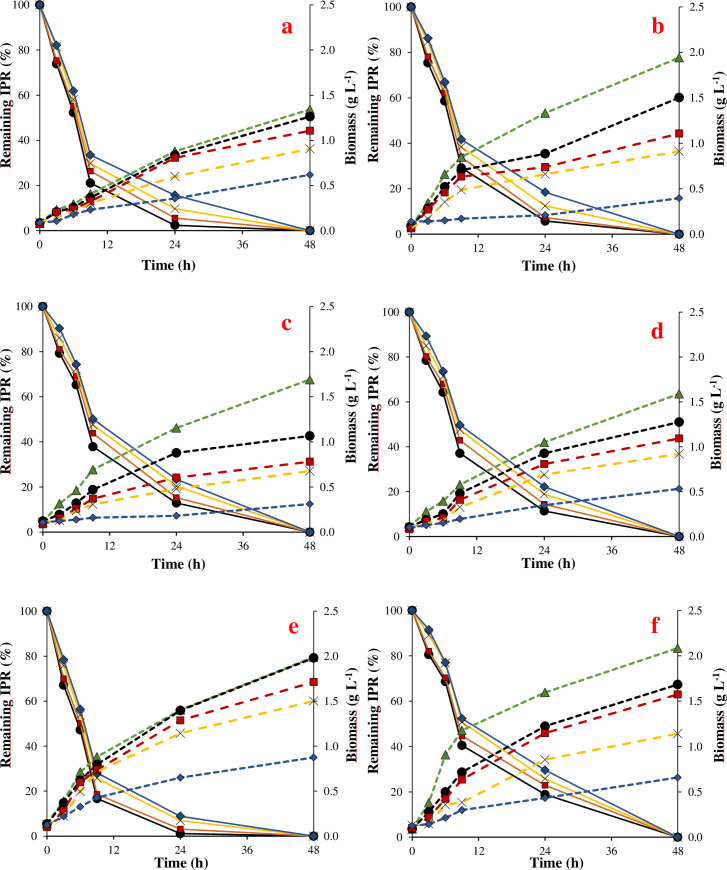
Remaining iprodione (IPR) (%) and biomass by C1 (a), C4 (b), C7 (c), C8 (d), C9 (3), C10 (f) strains at initial IPR concentration of 0, 10, 20, 50 and 100 mg L^-1^, evaluated during 120 h. (●) IPR 10 mg L^-1^; (◼) IPR 20 mg L^-1^; (x) IPR 50 mg L^-1^; (◆) IPR 100 mg L^-1^; (▲) Control without IPR. Continuous line: Remaining IPR (%); Dotted line: biomass (g L^-1^).

The Fig 3 shows that the biomass of bacteria exposed to IPR increased over time, though in smaller amounts than was observed for CHL. In the control treatment, a biomass between 1.43 and 2.38 g L^-1^ and μ max from 0.18 to 0.48 h^-1^ were observed, instead of a biomass between 0.78 and 1.80 g L^-1^ and μ max from 0.15 to 0.36 h^-1^ at 50 mg L^-1^ of IPR. Application of 100 mg L^-1^ IPR in the liquid medium caused a marked inhibition of microbial growth, with the biomass ranging between 0.34 to 1.06 g L^-1^ and μ max between 0.05 and 0.24 h^-1^ (Table 2). *Achromobacter* sp. strain C1 and *Pseudomonas* sp. strain C9 were the most tolerant strains to IPR in relation to the growth that was observed in the control treatment.

The removal of CHL showed significant differences (p ≤ 0.05) between the strains. Moreover, for all bacteria strains, as the contaminant concentration increased from 10 to 100 mg L^-1^, the removal of contaminants decreased. *Achromobacter* sp. strain C1 showed the best CHL removal with values between 29–56% after a 120 h incubation followed by the *Pseudomonas* sp. strain C4, which was able to remove between 28–42% of the CHL. Table 3 shows the kinetic parameters that were obtained for CHL removal. In this context, CHL removal by the C1 strain was characterized by a rate constant of 0.147–0.072 d^-1^ and T_1/2_ 4.7–9.7 d^-1^ in liquid medium treated with 10 and 100 mg L^-1^ CHL. This trend was closely followed by *Pseudomonas* sp. strain C4, *Achromobacter* sp. strain C7, and *Achromobacter* sp. strain C10, with a removal between 28–42%, 24–38%, and 19–37%, respectively. However, the lowest degradation was observed for *Pseudomonas* sp. strain C9 with CHL degradation between 14–20%, a rate constant between 0.047–0.032 d^-1^, and T_1/2_ 14–22 d^-1^.

**Table 3:** First-order kinetics parameter for chlorpyrifos (CHL) and iprodione (IPR) removal of strains C1, C4, C7, C8, C9 and C10 in liquid medium supplemented with 0–100 mg L^-1^ of pesticide individually.

	**Initial CHL concentration (mg L**^**-1)**^											
**Strain**	**10**			**20**			**50**			**100**		
	R	*k*	T_1/2_	R	*k*	T_1/2_	R	*k*	T_1/2_	R	*k*	T_1/2_
	(%)	(d^-1^)	(d)	(%)	(d^-1^)	(d)	(%)	(d^-1^)	(d)	(%)	(d^-1^)	(d)
**C1**	56.25 a	0.147	4.7	53.11 a	0.149	4.6	34.46 a	0.085	8.2	29.10 a	0.072	9.7
**C4**	41.80 b	0.113	6.1	38.04 b	0.097	7.2	28.60 b	0.069	10.0	28.04 a	0.061	11.4
**C7**	38.34 c	0.108	6.4	32.56 b	0.084	8.2	26.89 c	0.066	10.6	24.34 b	0.054	12.9
**C8**	29.75 d	0.067	10.4	19.70 cd	0.041	16.8	16.89 e	0.036	19.4	18.98 c	0.042	16.4
**C9**	19.82 e	0.047	14.7	15.29 d	0.037	18.6	15.88 f	0.030	22.8	13.57 d	0.032	21.9
**C10**	37.45 c	0.105	6.6	24.26 c	0.060	11.5	20.52 d	0.050	13.8	19.46 c	0.044	15.8
**Initial IPR concentration (mg L**^**-1)**^												
**Strain**	10			20			50			100		
	R	*k*	T_1/2_	R	*k*	T_1/2_	R	*k*	T_1/2_	R	*k*	T_1/2_
	(%)	(h^-1^)	(h)	(%)	(h^-1^)	(h)	(%)	(h)	(h)	(%)	(h^-1^)	(h)
**C1**	97.60 b	0.160	4	94.40 b	0.123	6	90.30 b	0.099	7	84.30 b	0.078	9
**C4**	94.20 c	0.121	6	92.70 c	0.112	6	87.50 c	0.089	8	81.50 c	0.072	10
**C7**	87.10 e	0.087	8	84.70 d	0.079	9	79.60 e	0.068	10	76.60 e	0.062	11
**C8**	88.60 d	0.092	8	85.60 d	0.082	8	81.10 d	0.071	10	77.80 d	0.065	11
**C9**	98.90 a	0.193	4	96.90 a	0.148	5	93.10 a	0.113	6	91.20 a	0.103	7
**C10**	81.10 f	0.070	10	77.10 e	0.062	11	74.20 f	0.058	12	70.40 f	0.052	13

Values of removal (% R) within a concentration with the same letter are not significantly different based on the Tukey test (α = 0.05), the average values of three replicates are presented (n = 3); R (%): removal of pesticides, *k*: rate constant, T_**1/2**_: half-life time, μ: specific growth rate.

The Fig 3 shows that the bacteria strains were able to efficiently remove IPR, with removals between 81–98% after 24 h of incubation; after this time IPR was not detected. The removal of IPR was significantly different between strains (p ≤ 0.05), with *Pseudomonas* sp. strain C9 showing the highest IPR removal (between 91.2–98.9%), even as the concentration of pesticide increased. According to the kinetic parameters shown in Table 3, the rate constant for IPR ranged from 4 to 13 h. The strain C9 showed the highest rate constant of 0.193 h^-1^ for IPR added at 10 mg L^-1^ and a maximum T_1/2_ of 7 h^-1^ when IPR was added at a concentration of 100 mg L^-1^. For *Achromobacter* sp. strain C10, the lowest IPR removal (70–81%) was observed after 24 h incubation and a T_1/2_ ranging from 11 to 13 h^-1^.

In both cases, no removal of CHL and IPR was observed in the flasks without inoculation by the selected bacteria strains (abiotic control).

The metabolites TCP and 3,5-DCA were produced during CHL and IPR removal, respectively. The metabolite concentrations increased over time and when the pesticide concentration increased. TCP production was highest when the liquid media was treated with *Achromobacter* sp. strain C1, detecting concentrations between 0.504–2.098 mg L^-1^ after 120 h incubation. For all the other strains, TCP concentrations ranged from 0.214 mg L^-1^ produced by *Pseudomonas* sp. strain C9 during the treatment of 10 mg L^-1^ CHL to 1.498 mg L^-1^ produced by *Pseudomonas* sp. strains C4 during the treatment of 100 mg L^-1^ CHL. In relation to IPR degradation, the results showed that the metabolite began to appear in the liquid medium at 9 h incubation. After 48 h incubation, the 3,5-DCA concentrations ranged between 0.210–0.384 mg L^-1^ and 0.648–0.945 mg L^-1^ after treatment of 10 and 100 mg L^-1^ IPR, respectively. Moreover, the highest 3,5-DCA concentrations were produced during the treatments of 20, 50, and 100 mg L^-1^ IPR by *Pseudomonas* sp. strain C9 (S3 Table).

## Discussion

Inappropriate pesticide management has resulted in their release into the environment, including water resources. Therefore, efforts to develop technologies, as well as BPS, which guarantee the effective treatment of pesticide residues have been made. The efficacy of BPS depends of its capacity to degrade pesticides, which is determined mainly by the microbial composition into the biomixture [7]. Screening for degrading microorganisms through an enrichment procedure from a pesticide-contaminated system allows for the selection of potential isolates with a high tolerance and maximal degrading activity [31]. In our study, the biomixture that was used for the isolation of microorganisms was repeatedly used for the treatment of pesticides. The amplicon analysis indicated the coexistence of representative phyla of bacteria into the biomixture. Similar with our observations, the predominant bacteria in biomixtures exposed to pesticides (atrazine, carborufan, diazinon, glyphosate and iprodione) were Proteobacteria, Actinobacteria and Firmicutes [9, 32]. All these phyla, together with Bacteroidete are reported as predominant bacteria in the soil [33, 34], which influence the microbial composition in the biomixture [32]. Among the identified phyla, there are a diversity of bacteria reported as pesticide degraders. Bacteria strains belonging from the phyla Protobacteria (*Achromobacter* sp.) and Actinobacteria (*Arthrobacter* sp.) are able to degrade the fungicide IPR [24] and the herbicide diuron [35], in the same way, Actinobacteria belonging from the *Streptomyces* genus are able to degrade CHL and diazinon [8], and Protobacteria such as *Xanthomonas* sp. and *Pseudomonas* sp. degrade CHL [36].

The use a biomixture allowed us to obtain ten strains that were tolerant, six of which were able to grow in the presence of CHL and IPR, which is indicative that both compounds could be used as carbon and energy sources, presumably via partial transformation reactions that can occur with different chemical classes of pesticides [18]. The characterization of isolated bacteria was conducted in different ways. Colony morphology and Gram staining are basic microbial techniques that are used to group bacteria. On the other hand, enzymatic characterization using the ApiZym test will allow us to know the production of different enzymes. It should be noted that some authors have highlighted bacteria-produced enzymes that were obtained from contaminated sites of high biotechnological, clinical, and industrial interest [25, 27]. In our study, strains C4, C8, and C9 presented the highest biochemical activity, being positive for at least 13 of the 24 enzymes that were tested, some of which hydrolase enzymes that are responsible for breaking ester and phosphate bonds, and amino groups. Moreover, four of the six tolerant strains were positive for the cellulolytic enzyme that is responsible for organic polymers hydrolysis in small and available carbohydrates [37].

Identification conducted by 16S rRNA gene sequencing, which is widely used to determine microorganism taxonomic positions, and MALDI-TOF/TOF MS, which identifies and classifies an organism according to the spectral profile of its ribosomal proteins, were concordant. A phylogenetic analysis of isolates showed a closer relation with the bacteria from genera *Pseudomonas*, *Achromobacter*, and *Rhodococcus*, which are known as metabolically active microorganisms that are capable of degrading different classes of pesticides, including CHL and IPR [9, 23, 38]. Use of MALDI-TOF/TOF MS has led to a new era of routine and rapid identification of different organisms, including environmental bacteria [11]. In our study, dendrograms generated by MALDI Biotyper were able to separate or assemble different strains in the same cluster, depending on the pesticide exposure, which could explain the effects on proteins due to environmental conditions [39].

The evaluation of CHL and IPR removal at increasing concentrations and different times by selected bacteria showed that both pesticides added at the highest concentration of 100 mg L^-1^ affected bacterial growth. Although CHL and IPR in liquid media could be used as a source of carbon and energy for growth [23, 24], there is not always a direct relationship between the consumed substrate that is converted into biomass [40]. Consumed substrate could be used for maintenance and not to produce new cells, which finally results in a decreased biomass yield [41]. On the other hand, the high pesticide concentration could limit the proliferation of degrading microorganisms. Specifically, during the CHL degradation, the metabolite TCP is produced, which is recognized by present antimicrobial properties [42]. Despite IPR or their metabolites, there is limited information on factors that may inhibit environmental bacteria [43], and their probably toxic effect has not been excluded.

According to our results, *Achromobacter* sp. C1 and *Pseudomonas* sp. C4 were the most tolerant strains to CHL, and *Achromobacter* sp. C1 and *Pseudomonas* sp. C9 were tolerant to IPR. These same strains were those that showed the highest pesticide removal. Studies reported that the strains isolated from biomixture degrade pesticides. For example, A*chromobacter xylosoxidans* strain CS5 removes endosulfan [44], a consortium that is formed by *Arthrobacter* sp. BS2 and *Achromobacter* sp. SP1 degrade diuron and their metabolite, 3,4-dichloroaniline [35]; *Pseudomonas* sp. and *Achromobacter* sp. isolated from agricultural soil degrade atrazine [38]; *Arthrobacter* sp. strain C1 and *Achromobacter* sp. strain C2 isolated from soil degrade IPR [24]; and *Pseudomonas* spp. has been commonly described as a CHL-degrader [23]. Despite most pesticide-degrading microorganisms have been isolated preferably from soil with a history of pesticide application, a biobed biomixture would be an appropriate option to obtain microorganism adapted to tolerate and degrade high loads of pesticides from different chemical classes.

*Achromobacter* sp. strain C1 evidenced the highest CHL removal, which is associated with the presence and activity of the enzyme alkaline phosphatase, as this enzyme is a phosphomonoesterase that regulates CHL degradation through the hydrolysis of O-P bonds [42]. Similarly, the presence of diverse enzymes in *Pseudomonas* sp. strain C4 could influence fast degradation and, thus, reduce the T_1/2_ required for pesticide reduction. Previous researchers have reported that CHL removal by bacteria occurred through the formation of metabolites, such as CHL-oxon, 3,5,6-trichloro-2-methoxypyridine, 2-chloro-6-hydroxypyridine, and TCP. In this study, the TCP produced was not metabolized by any strain; therefore, a slight increase over time and accumulation in the liquid medium was observed. A different response was reported by Briceño et al. [22] for Actinobacteria strains, which were able to metabolize TCP produced in liquid medium contaminated with 25 and 50 mg L^-1^ CHL. A lower concentration of TCP (0.46 mg L^-1^) was produced by *Streptomyces* sp. strain AC5, and its concentration decreased as a function of time, as the TCP produced was 10 times lower compared to that produced by *Streptomyces* sp. AC7 strain (4.32 mg L^-1^). TCP accumulation and the presence of chlorine atoms on the pyridinol ring caused a toxic effect on the microorganisms [42], which was the main influence in obtaining incomplete CHL removal in the time evaluated. Nonetheless, the removal of 10 mg L^-1^ and 20 mg L^-1^ of CHL was effectively performed by *Achromobacter* sp. strain C1, which required only a few days more to achieve complete CHL elimination. A study reported that *A*. *xylosoxidans* JCp4 was able to mineralize 100 mg L^-1^ CHL completely after 10 d with only a transient accumulation of TCP [45].

The appearance of 3,5-DCA, which is recognized as the major metabolite of IPR degradation at 9 h of incubation, was observed at concentrations lower than 0.5 mg L^-1^. The appearance of 3,5-DCA was coincident with the fastest decrease of IPR levels. After this time, the 3,5-DCA concentrations slightly increased, such that no IPR residues were found after 48 h incubation. In our study, a small amount of time was required for *Achromobacter* sp. strains C1 to remove 50% of the contaminant from liquid medium (T_1/2_ between 4–9 h), which signify the environmental adaptation of this bacteria being exposed to continued pesticide application in the biomixture that was used for their isolation. Although IPR is a common fungicide that is frequently used in crops and has a classification of “probable carcinogen to humans,” treatment to eliminate IPR by using microorganisms has been poorly studied.

Mercadier et al. [46] isolated from soil adapted to IPR three bacterial strains identified as *Pseudomonas fluorescens*, *Pseudomonas* sp., and *Pseudomonas paucimobilis*. These strains were responsible of the IPR transformation to 3,5-DCA occurs via 3,5-dichlorophenyl-carboxamide (metabolite I), which was subsequently transformed to 3,5-dichlorophenylurea-acetate (metabolite II). Later, Campos et al. [24] isolated and characterized two bacterial cultures (C2.7 and A1.4) from acidic pristine soil that degraded IPR and 3,5-DCA. Subsequently by molecular fingerprinting revealed that C2.7 was composed of two strains, identified via cloning as *Arthrobacter* sp. (strain C1) and *Achromobacter* sp. (strain C2), whereas A1.4 was pure and it was identified as *Pseudomonas* sp. Degradation studies with the purified isolates *Arthrobacter* sp. strain C1, *Achromobacter* sp. strain C2 and their combination in minimal and rich media showed that *Arthrobacter* sp. strain C1 was the key iprodione-degrader that obtained a complete degradation within 8 and 24 h culture, and this strain maintained its degrading capacity in a wide range of temperatures and pH, whereas *Achromobacter* sp. strain C2 was only able to slowly co-metabolize iprodione in a rich medium after 240 h [24]. Posteriorly, the authors reported that IPR removal occur via initial hydrolysis to isopropylamine and metabolite I and then to metabolite II before being hydrolysed to 3,5-DCA and, probably, glycine. Yang et al. [47] reported that IPR was degraded through the typical pathway by a novel amidase enzyme present in the *Paenarthrobacter* sp. strain YJN-5. According with our results, the isolated bacteria adapted to IPR could constitute a source of hydrolytic enzymes (e.g. esterase, phosphatase) responsible for IPR transformation. However, we did not investigate which enzyme could be involved in the IPR degradation. Therefore, futures assay must be addressed to elucidate the biochemical mechanisms.

For the effective use of microorganisms in bioremediation processes, it is extremely important to determine their potential for the removal of pesticides in liquid media under optimal conditions [2]. However, the source of isolation constitutes an important factor that will also influence the removal levels achieved. This study reports the isolation and characterization of bacteria isolated from BPS, which is increasingly incorporated as a pesticide treatment system. Although future assays are required to optimize pesticide removal by our isolated bacteria, they constitute potential microorganisms to use as inoculum in BPS, as well as for use in bioreactors for the treatment of wastewater containing CHL and IPR.

## Conclusion

This work reports the amplicon characterization of a BPS repeatedly used for the treatment of pesticides. This characterization informed that the microbial community composition is mainly formed by the phyla Proteobacteria, Firmicutes, Bacteroidetes and Actinobacteria. A total of six different bacteria isolated from the biomixture were tolerant to the pesticides. Moreover, these bacteria were capable of degrading chlorpyrifos (CHL) and iprodione (IPR). Given the identification of these strains and their ability to remove contaminants, *Achromobacter* sp. strain C1 and *Pseudomonas* sp. strain C9 appear to be promising microorganisms for the treatment of matrices contaminated with CHL, IPR, or their mixture.

## Supporting information

S1 Table Physicochemical characterization for the tested commercial pesticides. (DOCX)

S2 Table Phenotypic features and biochemical characteristics of pesticide-tolerant bacteria isolated from biopurificaction system (BPS). (DOCX)

S3 Table Production of 3,5,6-trichlo-2-pyridinol (TCP) and 3,5-DCA by strains C1, C4, C7, C8, C9 and C10 in liquid medium supplemented with 0–100 mg L^-1^ of chlorpyrifos (CHL) and iprodione (IPR) individually. (DOCX)

S1 Fig Electron scan micrographs of cells morphology of C4 (a), C8 (b) and C10 (c) strains isolated by enrichment culture from a biomixture of a biopurification system treated repeatedly with pesticides.
(DOCX)

S2 Fig Phylogenetic tree constructed by the neighbor-joining method based on 16S rRNA sequences of studied C1, C4, C7, C8, C9, C10 strains and related ones. (DOCX)

S3 Fig Dendrogram obtained by MALDI Biotyper Compass 4.1 software (Bruker Daltonics, Bremen, Germany) of C1, C4, C7, C8, C9, C10 strains after enrichment with CHL and IPR of liquid cultures. (DOCX)
