# Supplementary material for: Pesticide-tolerant bacteria isolated from a biopurification system to remove commonly used pesticides to protect water resources
Source: PLoS One. 2020 Jun 29;15(6):e0234865. doi: 10.1371/journal.pone.0234865 (PMC7324069; doi:10.1371/journal.pone.0234865)
Supplement: S1 Table — (DOCX) [file pone.0234865.s001.docx]

**Supplementary Data Table 1.**

| **Pesticide** | **Commercial product** | **Water solubility**  **(mg L^-1^)** | **Molecular weight**  **(g mol^-1^)** | **T_1/2_**  **(d)** | **GUS** | **Koc** |
| --- | --- | --- | --- | --- | --- | --- |
| Chlorpyrifos | Troya 4 EC | 1.05 | 350.58 | 50 | 0.17 | 8151 |
| Iprodione | Rovral 50 WP | 6.80 | 330.17 | 36 | 0.58 | 700 |

Solubility in water at 20 ºC; T_1/2_: Time half-life, GUS: Groundwater Ubiquity Score; Koc: Adsorption coefficient.
