# Supplementary material for: Pesticide-tolerant bacteria isolated from a biopurification system to remove commonly used pesticides to protect water resources
Source: PLoS One. 2020 Jun 29;15(6):e0234865. doi: 10.1371/journal.pone.0234865 (PMC7324069; doi:10.1371/journal.pone.0234865)
Supplement: S2 Table — (DOCX) [file pone.0234865.s002.docx]

**Supplementary Data Table 2.**

|  | **Response of strains** | | | | | |
| --- | --- | --- | --- | --- | --- | --- |
|  | **C1** | **C4** | **C7** | **C8** | **C9** | **C10** |
| **Color of colonies** | Cream | Cream | White | Dark-Cream | Cream | Cream |
| **Morphology** | Bacillus | Bacillus | Bacillus | Coccus | Bacillus | Bacillus |
| **Gram staining** | **-** | **-** | **-** | **+** | **-** | **-** |
| **Enzyme*** | **C1** | **C4** | **C7** | **C8** | **C9** | **C10** |
| Control | **-** | **-** | **-** | **-** | **-** | **-** |
| Alkaline phosphatase | + | - | + | + | + | + |
| Esterase (C4) | + | + | + | + | + | + |
| Esterase lipase (C8) | - | + | - | + | + | - |
| Lipase (C14) | - | + | - | + | - | - |
| Leucine aminopeptidase | + | + | + | + | + | + |
| Valine aminopeptidase | + | + | - | + | + | + |
| Cystine aminopeptidase | - | - | - | + | - | - |
| Trypsin | - | + | - | - | + | - |
| α- Chymotrypsin | - | - | - | + | - | - |
| Acid phosphatase | + | + | + | + | + | + |
| Naphthol-AS-BI-phosphohydrolase | + | + | + | + | + | + |
| α-Galactosidase | - | - | - | - | - | - |
| β- Galactosidase | - | - | - | - | - | - |
| β-Glucuronidase | - | - | - | - | - | - |
| α- Glucosidase | - | - | - | + | - | - |
| β- Glucosidase | - | - | - | + | - | - |
| N-acetyl-β- Glucosaminidase | - | - | - | - | - | - |
| α- Mannosidase | - | - | - | - | - | - |
| α- Fucosidase | - | - | - | - | - | - |
| **Extracellular hydrolase activity ^#^** | **C1** | **C4** | **C7** | **C8** | **C9** | **C10** |
| Amylolysic (starch 0.4%) | + | + | + | + | + | + |
| Cellulolytic (CMC 0.4%) | + | + | - | + | + | - |
| Lipolytic (tween 80%) | + | + | + | + | + | + |
| Proteolytic (milk 30%) | - | + | - | - | + | - |
| Proteolytic (gelatin 1%) | - | + | - | - | + | - |

+ : Positive reaction, - : Negative reaction; *Analysed by API ZYM kit; ^#^ response on R2A agar.
