# Supplementary material for: Pesticide-tolerant bacteria isolated from a biopurification system to remove commonly used pesticides to protect water resources
Source: PLoS One. 2020 Jun 29;15(6):e0234865. doi: 10.1371/journal.pone.0234865 (PMC7324069; doi:10.1371/journal.pone.0234865)
Supplement: S3 Table — (DOCX) [file pone.0234865.s003.docx]

**Supplementary Data Table 3.**

| **Strains** |  | **Initial CHL concentration (mg L^-1^)** | | | |  | **Initial IPR concentration (mg L^-1^)** | | | | |
| --- | --- | --- | --- | --- | --- | --- | --- | --- | --- | --- | --- |
|  | **Time** | **10** | **20** | **50** | **100** |  | **Time** | **10** | **20** | **50** | **100** |
|  | **(h)** | **TCP (mg L^-1^)** | | | |  | **(h)** | **3,5-DCA (mg L^-1^)** | | | |
| C1 | 24 | 0.17±0.01 | 0.36±0.02 | 0.82±0.02 | 1.61±0.02 |  | 9 | 0.21±0.00 | 0.35±0.01 | 0.29±0.01 | 0.47±0.01 |
|  | 72 | 0.22±0.29 | 0.41±0.01 | 0.94±0.01 | 1.84±0.03 |  | 24 | 0.24±0.05 | 0.36±0.02 | 0.45±0.02 | 0.65±0.01 |
|  | 120 | 0.50±0.02 | 0.69±0.01 | 1.62±0.02 | 2.10±0.02 |  | 48 | 0.38±0.00 | 0.42±0.04 | 0.52±0.06 | 0.87±0.10 |
| C4 | 24 | 0.12±0.02 | 0.32±0.02 | 0.80±0.02 | 1.40±0.02 |  | 9 | 0.15±0.03 | 0.29±0.01 | 0.26±0.01 | 0.35±0.01 |
|  | 72 | 0.14±0.01 | 0.33±0.01 | 0.88±0.04 | 1.47±0.04 |  | 24 | 0.20±0.06 | 0.34±0.01 | 0.41±0.06 | 0.59±0.06 |
|  | 120 | 0.34±0.01 | 0.52±0.00 | 1.48±0.08 | 1.50±0.04 |  | 48 | 0.34±0.01 | 0.41±0.07 | 0.49±0.02 | 0.86±0.06 |
| C7 | 24 | 0.08±0.02 | 0.28±0.02 | 0.74±0.02 | 1.42±0.02 |  | 9 | 0.32±0.03 | 0.16±0.03 | 0.19±0.07 | 0.27±0.05 |
|  | 72 | 0.10±0.00 | 0.30±0.01 | 0.77±0.02 | 1.46±0.04 |  | 24 | 0.18±0.02 | 0.26±0.04 | 0.29±0.04 | 0.41±0.07 |
|  | 120 | 0.29±0.01 | 0.48±0.02 | 1.27±0.04 | 1.47±0.02 |  | 48 | 0.30±0.02 | 0.35±0.01 | 0.37±0.02 | 0.75±0.13 |
| C8 | 24 | 0.08±0.02 | 0.27±0.02 | 0.66±0.02 | 1.38±0.02 |  | 9 | 0.15±0.02 | 0.20±0.03 | 0.22±0.04 | 0.35±0.01 |
|  | 72 | 0.07±0.01 | 0.26±0.01 | 0.73±0.04 | 1.46±0.03 |  | 24 | 0.20±0.00 | 0.32±0.05 | 0.35±0.05 | 0.50±0.02 |
|  | 120 | 0.24±0.00 | 0.42±0.02 | 0.96±0.03 | 1.45±0.00 |  | 48 | 0.31±0.04 | 0.39±0.01 | 0.42±0.04 | 0.85±0.08 |
| C9 | 24 | 0.07±0.02 | 0.26±0.02 | 0.61±0.02 | 1.36±0.03 |  | 9 | 0.25±0.00 | 0.38±0.03 | 0.37±0.03 | 0.47±0.08 |
|  | 72 | 0.06±0.01 | 0.25±0.01 | 0.78±0.04 | 1.38±0.04 |  | 24 | 0.26±0.02 | 0.38±0.01 | 0.47±0.04 | 0.70±0.04 |
|  | 120 | 0.21±0.00 | 0.47±0.01 | 0.91±0.01 | 1.40±0.00 |  | 48 | 0.31±0.00 | 0.49±0.01 | 0.59±0.03 | 0.95±0.00 |
| C10 | 24 | 0.06±0.02 | 0.25±0.02 | 0.74±0.02 | 1.56±0.02 |  | 9 | 0.07±0.01 | 0.10±0.02 | 0.10±0.00 | 0.22±0.01 |
|  | 96 | 0.07±0.03 | 0.26±0.02 | 0.83±0.01 | 1.45±0.08 |  | 24 | 0.17±0.06 | 0.12±0.04 | 0.22±0.03 | 0.39±0.02 |
|  | 120 | 0.28±0.00 | 0.47±0.01 | 0.98±0.01 | 1.38±0.00 |  | 48 | 0.21±0.05 | 0.25±0.00 | 0.35±0.00 | 0.65±0.05 |

The average values and the standard error are presented (n= 3)
