# Supplementary figures and images for: Pesticide-tolerant bacteria isolated from a biopurification system to remove commonly used pesticides to protect water resources
Source: PLoS One. 2020 Jun 29;15(6):e0234865. doi: 10.1371/journal.pone.0234865 (PMC7324069; doi:10.1371/journal.pone.0234865)

*
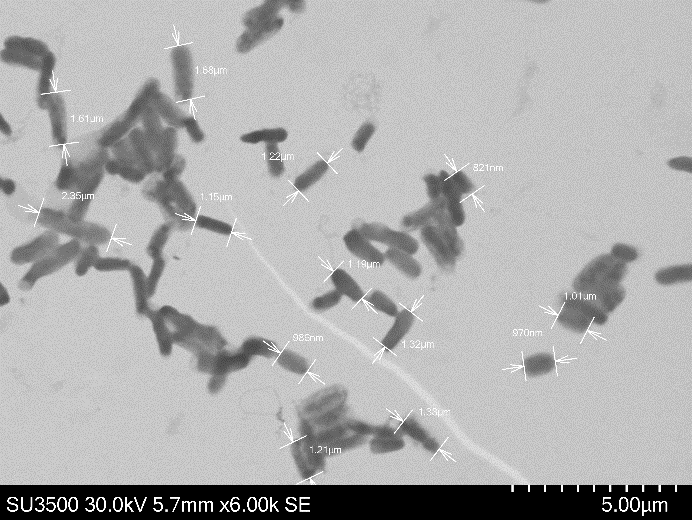
*

**a**

**a**

*
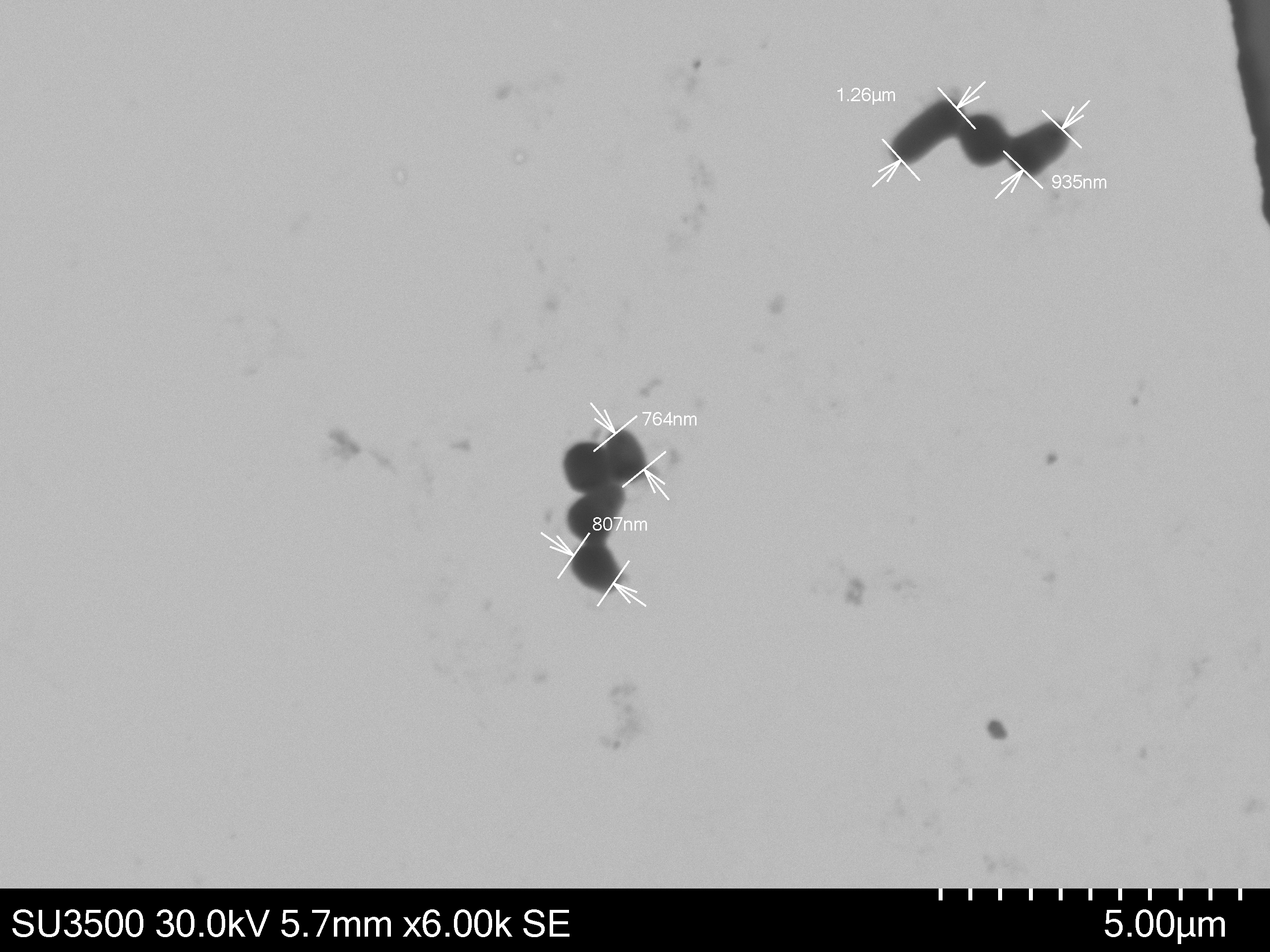
*

**b**

**b**


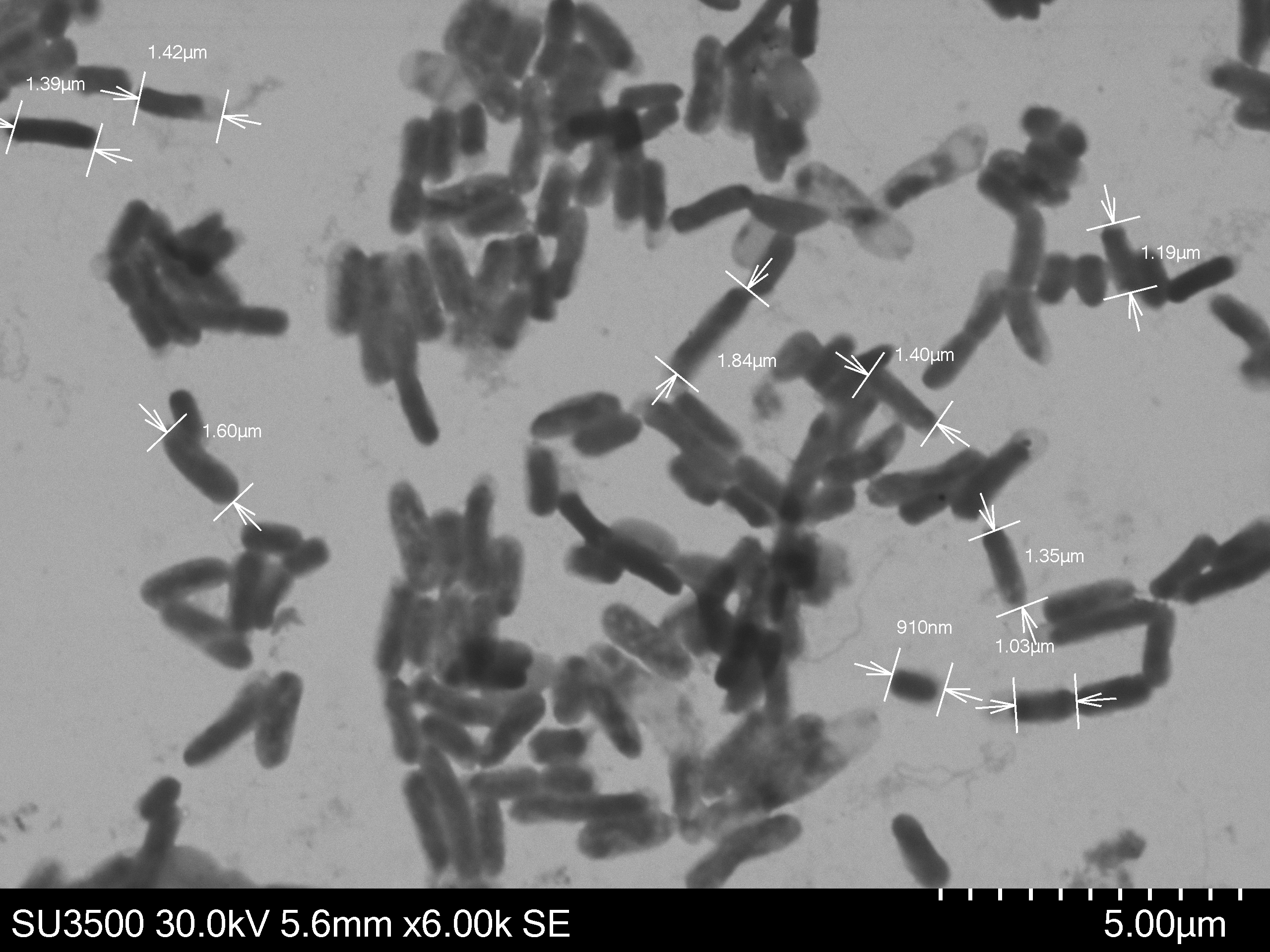


**c**

**S2 Fig 1.**

**c**

Supplement: S1 Fig — Electron scan micrographs of cells morphology of C4 (a), C8 (b) and C10 (c) strains isolated by enrichment culture from a biomixture of a biopurification system treated repeatedly with pesticides. (DOCX) [file pone.0234865.s004.docx]

**S2 Fig 3.**


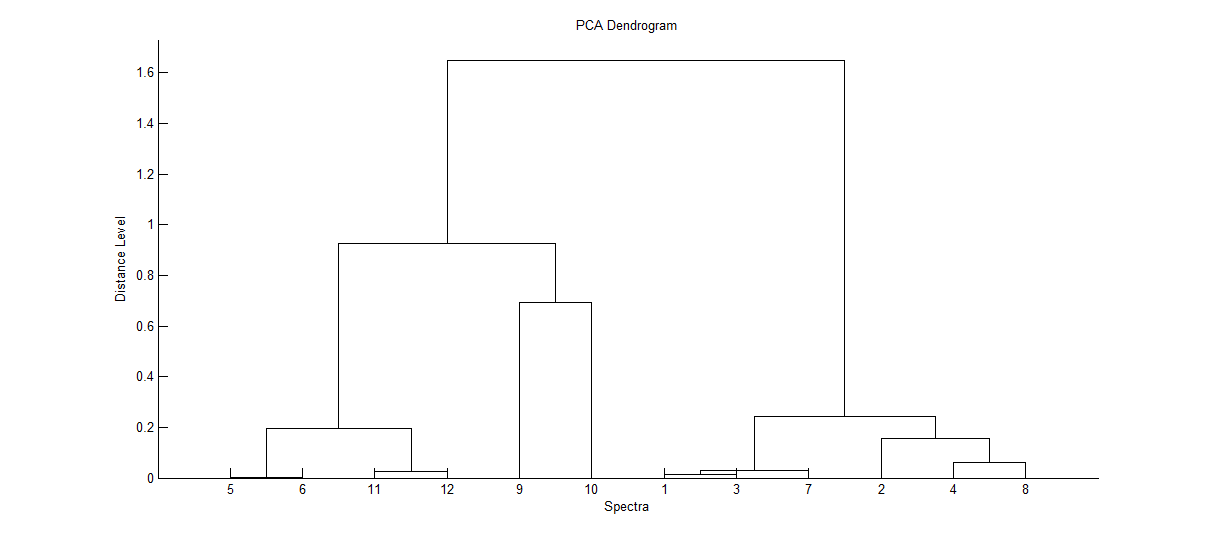

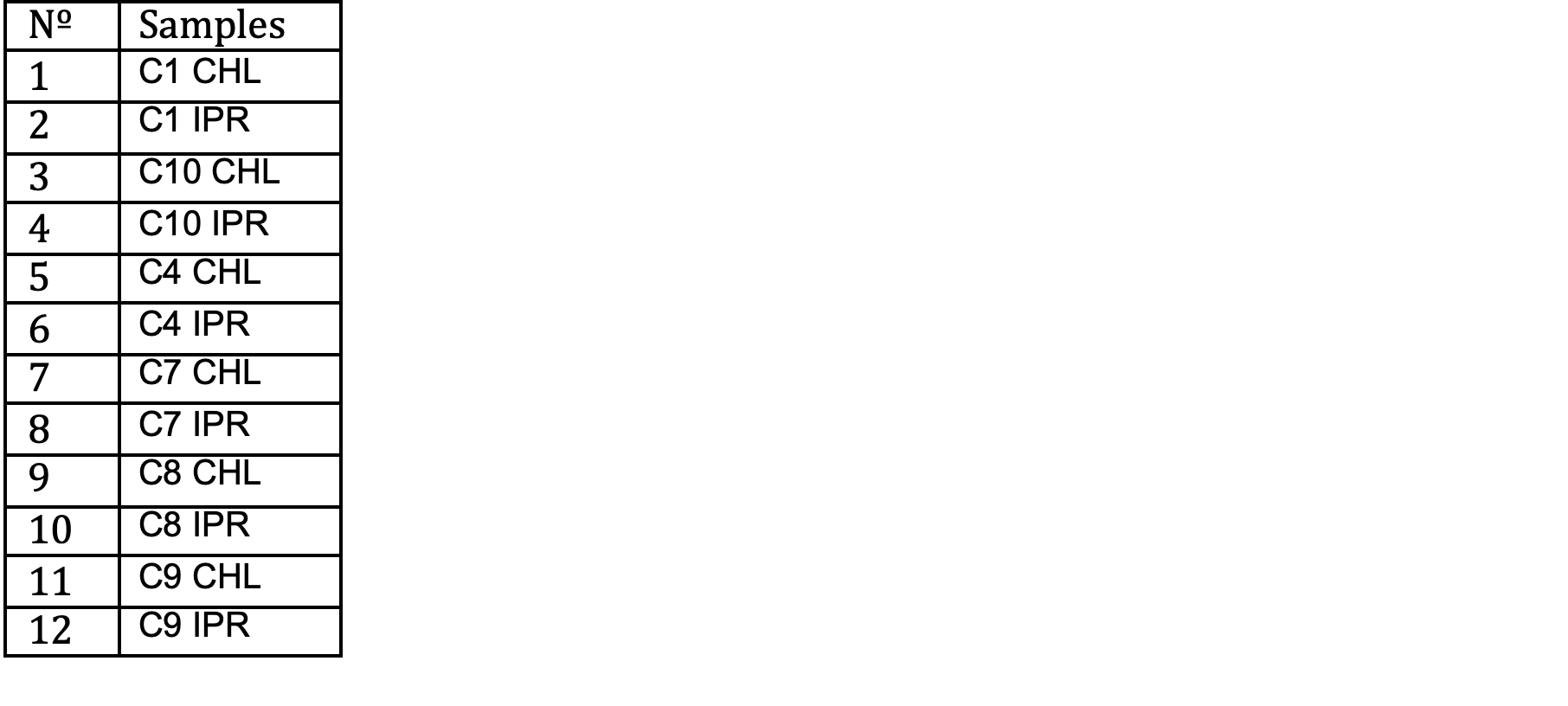

Supplement: S3 Fig — (DOCX) [file pone.0234865.s006.docx]
