# Supplementary material for: Pesticide-tolerant bacteria isolated from a biopurification system to remove commonly used pesticides to protect water resources
Source: PLoS One. 2020 Jun 29;15(6):e0234865. doi: 10.1371/journal.pone.0234865 (PMC7324069; doi:10.1371/journal.pone.0234865)
Supplement: S2 Fig — (DOCX) [file pone.0234865.s005.docx]

**S2 Fig 2.**
